# Maternal vitamin B_12_ in mice positively regulates bone, but not muscle mass and strength in post-weaning and mature offspring

**DOI:** 10.1152/ajpregu.00355.2020

**Published:** 2021-03-24

**Authors:** Parminder Singh, Svetalana Telnova, Bin Zhou, Abdalla D. Mohamed, Vanessa De Mello, Henning Wackerhage, X. Edward Guo, Amulya K. Panda, Vijay K. Yadav

**Affiliations:** ^1^Metabolic Research Laboratory, National Institute of Immunology, New Delhi, India; ^2^Wellcome Trust Sanger Institute, Cambridge, United Kingdom; ^3^Bone Biomechanics Laboratory, Columbia University, New York, New York; ^4^Cancer Therapeutics Unit, The Institute of Cancer Research, London, United Kingdom; ^5^University of Aberdeen, Aberdeen, United Kingdom; ^6^Exercise Biology Group, Faculty of Sport and Health Sciences, Technical University of Munich, Munich, Germany; ^7^Department of Genetics and Development, Columbia University, New York, New York

**Keywords:** bone, gut, muscle, osteoblasts, vitamin B_12_

## Abstract

Vitamin B_12_ deficiency has been shown to affect bone mass in rodents and negatively impact bone formation in humans. In this study using mouse models, we define the effect of B_12_ supplementation in the wild-type mother and B_12_ deficiency in a mouse genetic model (*Gif*^−/−^ mice) during gestation on bone and muscle architecture and mechanical properties in the offspring. Analysis of bones from 4-wk-old offspring of the wild-type mother following vehicle or B_12_ supplementation during gestation (from *embryonic day 0.5* to *20.5*) showed an increase in bone mass caused by an isolated increase in bone formation in the B_12_-supplemented group compared with vehicle controls. Analysis of the effect of B_12_ deficiency in the mother in a mouse genetic model (*Gif*^−/−^ mice) on the long bone architecture of the offspring showed a compromised cortical and trabecular bone mass, which was completely prevented by a single injection of B_12_ in the B_12_-deficient *Gif*^−/−^ mothers. Biomechanical analysis of long bones of the offspring born from B_12_-supplemented wild-type mothers showed an increase in bone strength, and conversely, offspring born from B_12_-deficient *Gif*^−/−^ mothers revealed a compromised bone strength, which could be rescued by a single injection of B_12_ in the B_12_-deficient *Gif*^−/−^ mother. Muscle structure and function analysis however revealed no significant effect on muscle mass, structure, and grip strength of B_12_ deficiency or supplementation in *Gif*^−/−^ mice compared with littermate controls. Together, these results demonstrate the beneficial effect of maternally derived B_12_ in the regulation of bone structure and function in the offspring.

## INTRODUCTION

Mammalian bone and skeletal muscles are intricately connected and coordinate locomotion (1). The biomechanical strength of the vertebrate skeleton is determined by the changes in structure and functional properties of either bone or skeletal muscle or both (1). Healthy bone strength depends on the overall geometry, thickness, and porosity of the bone tissue (2). These structural changes in the bone tissue are coordinated by the localized changes in the bone remodeling happening throughout the skeleton (3). A healthy bone structure also depends on the changes in the structure and mass of the skeletal muscles that surround the bone (4). Skeletal muscle is the organ that converts the chemical energy in nutrients into mechanical and heat energy and plays an important role in determining localized changes in bone mass (5). Skeletal muscle is composed of muscle fibers [∼400,000–900,000 in human vastus lateralis that can contain thousands of nuclei and can be up to 20-cm long (6)]. Deterioration in the quality of bone and skeletal muscle tissues of the skeleton results in an increased risk of fractures, yet the factors regulating this process are still not fully understood (7).

Vitamin B_12_ (B_12_) is the most complex vitamin discovered to date, which is essential for humans and other animals (8, 9). Although animals need B_12_ to support their functions, animal cells cannot synthesize B_12_, which can only be synthesized by certain bacterial species (10). The gastrointestinal (GI) tract of many animals such as fish, ruminants, and chicken provide a favorable environment for the growth of the B_12_-synthesizing bacteria; B_12_ following its synthesis by these bacteria is absorbed in the circulation and stored in their tissues, primarily in the liver (11). The human and rodent GI tract, unlike many animals, cannot support the growth of the B_12_-synthesizing bacteria and therefore we obtain B_12_ through the dietary intake of products of animal origin such as meat and poultry (12).

The absorption of B_12_, either diet derived or commensal bacteria synthesized, from the gut requires a stomach-synthesized protein gastric intrinsic factor (Gif) (9). Once acquired, animals can recycle B_12_ to maintain cellular processes dependent on B_12_ (9). Vitamin B_12_ deficiency could arise, among other conditions, in the context of poor maternal nutrition, aging, vegetarian lifestyle, or gastric bypass surgery that removes part of the stomach that secretes Gif protein (13, 14). In humans, B_12_ deficiency leads to compromised bone quality and increased risk of fractures (15, 16), yet the impact of alterations in maternal B_12_ levels on bone architectural and biomechanical properties has never been investigated. Moreover, the effect of alterations in maternal B_12_ levels on muscle architecture has not been studied earlier.

Recently, we generated a mouse genetic model of B_12_ deficiency by ablating the protein Gif that is essential for B_12_ absorption from the gut. In this mouse genetic model the consequences of B_12_ deficiency on the growth is precipitated only in the second (F2) generation *Gif*^−/−^ mice and not the first (F1) generation (17). We showed that F2 *Gif*^−/−^ mice have normal bone length, bone weight, calcium levels, skeletal mineralization at birth, and normal growth up until 21 days of age, but after that, they develop low bone mass due to an abrogation in the B_12_-taurine-bone pathway that operates through the liver (17). Analysis of vertebra from *Gif*^−/−^ mice showed that the mutant mice of the second generation but not the first generation display low bone mass at 8 wk of age through histology and histomorphometry in trabeculae-rich vertebra, but we did not analyze changes in their bone architecture and strength in cortical-rich long bones and muscle architecture, mass, or function (17). Moreover, it is not known whether increasing B_12_ levels in the mother during gestation in the wild-type (WT) mice affect bone mass in the offspring during the peripubertal period. The purpose of this study was to characterize changes in bone and muscle properties in the offspring born from B_12_-supplemented and B_12_-deficient mothers. Our results show that the effect of B_12_ supplementation or deficiency in the mother specifically affects bone, but not muscle mass and function.

## MATERIALS AND METHODS

### Animals

All procedures performed on mice were approved by the ethics committees of the Wellcome Trust Sanger Institute, United Kingdom Home Office (Project license PPL80/2479) and National Institute of Immunology. *Gif*-deficient (*Gif^tm1a(KOMP)Wtsi/tm1a(KOMP)Wtsi^*, abbreviated to *Gif^tm1a/tm1a^* in this report) mice carry a knockout-first allele in which a promoter-less cassette including LacZ and neo genes were inserted in intron 3–4 of the *Gif* gene. *Gif* mice were generated using C57Bl/6N ES cells and were on pure C57Bl/6N background. WT C57Bl/6J mice were obtained from the in-bred wild-type colonies maintained at the National Institute of Immunology research support facility.

### Cyanocobalamin Treatment in WT Mice during Pregnancy

We subjected 6-wk-old virgin WT female mice (*n* = 4 per group) for timed mating with WT males. The day of detection of the plug was designated as 0.5 days postcoitum (dpc) and animals either received no treatment or were injected subcutaneously with 20 or 200 µg of CN-B_12_ from 0.5 dpc. These doses of B_12_ were chosen based on a pilot experiment which showed that subcutaneous injections of B_12_ at 20 and 200 µg for a week were sufficient to increase levels of serum taurine through which B_12_ mediates its effect on osteoblast proliferation and bone formation (17). We dissolved CN-B_12_ in saline (0.9% sodium chloride) (Sigma) and gave 200 µL of this solution subcutaneously to the pregnant mice. *n* = 7 ± 1 pups were born per female, and from the pups born *n* = 7–10 females were used for the analysis. In our previous study, we have shown that both males and females show low bone mass following B_12_ deficiency in the mouse genetic model and therefore we only analyzed data from the female mice to minimize the number of animals used in our studies. Males and females were separated at 21 days of age and housed in separate cages. Offspring were weighed every 5 days after birth until culling through CO_2_ inhalation (0.2%) and the skeleton was fixed in 4% neutral buffered formalin for histology and microcomputed tomography (µCT) analyses, while the right femur was frozen at −20°C in saline-soaked cotton gauze for biomechanical testing.

### Breeding Strategy for Generation of WT, *Gif^−/−^*_(F1)_, and *Gif^−/−^*_(F2)_ Mice

Generation of B_12_-deficient mice has been described previously (17). Briefly, to generate B_12_-deficient animals, we first crossed *Gif*^+/−^ female mice with *Gif*^+/−^ male mice, yielding first-generation *Gif*^+/+^ [*Gif*^+/+^_(F1)_] and *Gif*^−/−^_(F1)_ mice (17). Serum B_12_ levels in *Gif*^−/−^_(F1)_ offspring are reduced ∼20-fold compared with *Gif*^+/+^_(F1)_, yet these mice still harbored detectable levels of serum B_12_. To further deplete B_12_ levels in the offspring, we next crossed *Gif*^−/−^_(F1)_ females with *Gif*^−/−^_(F1)_ males to generate second-generation *Gif*^−/−^_(F2)_ mice; WT control mice were generated by crossing *Gif*^+/+^_(F1)_ females with *Gif*^+/+^_(F1)_ males. Serum B_12_ levels in *Gif*^−/−^_(F2)_ mice are <45 ng/L (i.e., below the limit of detection of the assay) compared with >11,000 ng/L in WT mice (17). Changes in B_12_ levels do not affect the survival of the offspring. F1 *Gif*^−/−^ mice that have a 20-fold reduction in serum B_12_ levels and F2 *Gif*^−/−^ that have B_12_ levels below the limit of detection of assay compared to WT mice survive well till the time point we have analyzed, that is, 1 yr of age. However, changes in B_12_ levels do affect fertility. Litter size from *Gif*^+/+^_(F1)_ mothers crossed with *Gif*^+/+^_(F1)_ fathers was 7 ± 1, whereas from *Gif*^−/−^_(F1)_ mothers crossed with *Gif*^−/−^_(F1)_ fathers was 5 ± 1. *Gif*^−/−^_(F2)_ females display atrophied uterus and other genital organs and are infertile, whereas males have smaller testes (V. K. Yadav, unpublished observations). Offspring were separated from their mothers at 24–26 days of age, and females and males were kept in separate cages until euthanasia and analysis. We have shown earlier that male and female B_12_-deficient offspring show low bone mass (17) and thus we only used females to minimize the number of animals used in our study. *n* = 8–10 females were used for analysis except in the case of muscle studies where *n* = 3 was used per group.

### Cyanocobalamin or Vehicle Treatment in WT and Gif^−/−^_(F1)_ Mice

We subjected 6-wk-old virgin *Gif*^−/−^_(F1)_ female mice (*n* = 4 per group) for timed mating with *Gif*^−/−^_(F1)_ male mice or WT females (*n* = 4) with WT males. The two genotypes for mating were derived from the littermates born from *Gif*^+/−^ parents. The day of detection of the plug was designated as 0.5 dpc and pregnant WT animals received the vehicle on 12.5 dpc. *Gif*^−/−^_(F1)_ pregnant animals were divided into two groups, one group received vehicle, whereas the other group was injected subcutaneously with 200 µg of CN-B_12_ on 12.5 dpc. The offspring from the above groups are labeled as WT_(VEH)_, *Gif*^−/−^_(F2:VEH)_, and *Gif*^−/−^_(F2:B12)_. We dissolved CN-B_12_ in saline (0.9% sodium chloride) (Sigma) and gave 200 µL of this solution subcutaneously to the pregnant mice. Offspring were weighed every 5 days after birth until culling through CO_2_ inhalation (0.2%) and the skeleton was fixed in 4% neutral buffered formalin for histology and µCT analyses, while the right femur was frozen at −20°C in saline-soaked cotton gauze.

### Microcomputed Tomography Analysis

Trabecular bone and cortical architecture of the proximal tibia were assessed using a µCT system (Skyscan 1172) as described previously (17). Tibia bone specimen was stabilized with gauze in a 2-mL centrifuge tube filled with 70% ethanol and fastened in the specimen holder of the µCT scanner. One hundred µCT slices, corresponding to a 1.05-mm region distal from the growth plate, were acquired at an isotropic spatial resolution of 10.5 µm. A global thresholding technique was applied to binarize gray-scale µCT images where the minimum between the bone and bone marrow peaks in the voxel gray value histogram was chosen as the threshold value. The trabecular bone compartment was segmented by a semiautomatic contouring method and subjected to a model-independent morphological analysis (18) by the standard software provided by the manufacturer of the µCT scanner. Three-dimensional (3D) morphological parameters, including model-independent measures by distance transformation (DT) of bone volume fraction (BV/TV), trabecular thickness (Tb.Th*), trabecular number (Tb.N*), trabecular separation (Tb.Sp*), and connectivity density (Conn.D) were evaluated. The Conn.D is a quantitative description of the trabecular connection (19, 20).

### Histology and Histomorphometry of Vertebra and Long Bones

Skeletal processing and histological and histomorphometric analysis were performed as described previously (21). For histological analysis, mice were euthanized at indicated ages. Briefly, for assessment of dynamic histomorphometric indices, mice were injected with calcein 2 and 4 days before euthanasia according to the standard calcein double-labeling procedure described previously (17). After euthanasia, internal organs were removed from the animals and the whole skeleton was pinned to a thermocol board and immersed in 4% neutral buffered formalin for 12–14 h at room temperature. After fixation the skeleton was cut into a vertebral column (lumbar vertebra 1–5 with associated muscles) or long bone (with associated muscles) for processing and embedding. Undecalcified bones were dehydrated in a series of ethanol, embedded in methyl methacrylate, and 5-μm sections were prepared on a rotation microtome (Leica Inc.) as described previously (17). Sections were stained with 1% toluidine blue (osteoblast parameters), or Von Kossa/Van Gieson reagent (distinguishes mineralized bone matrix in black and nonmineralized bone matrix in red color) or tartrate-resistant alkaline phosphatase (TRAP, osteoclast parameters) stains and evaluated using a Zeiss microscope (Carl Zeiss, Jena, Germany). Histomorphometrical analysis was performed on tibiae and vertebrae according to the American Society for Bone and Mineral Research (ASBMR) standards (22) using the OsteoMeasure Analysis System (Osteometrix, Atlanta, GA). For BV/TV% analysis, Von Kossa/Van Gieson-stained sections were imaged at ×5 magnification and analysis of bone volume over total volume was performed using Image J software. For osteoblast parameters, toluidine blue-stained sections were visualized at ×20 magnification and analyses were performed in the secondary spongiosa in at least 15 fields per vertebra or long bone section. For osteoclast parameters, TRAP-stained sections were visualized at ×20 magnification and analyses were performed in the secondary spongiosa in at least 15 fields per vertebra or long bone section. For bone formation analysis sections were cleared in xylene, mounted in DPX, and visualized under ultraviolet light at ×40 magnification and analysis was performed to detect bone formation fronts.

### Biomechanical Strength Testing of Femur

Biomechanical analysis of the bones was performed as described previously (23). Briefly, the right femur was collected from mice at indicated ages at the time of euthanasia and frozen at −20°C in saline-soaked cotton gauze for biomechanical testing. Three-point bending of the femur at the midshaft site was performed to assess biomechanical strength properties on these bones using a materials testing system (Model 4442 Universal Testing System; Instron Corp., Canton, MA) and specialized software program (Instron Series IX Automated Materials Tester, v. 8.15.00; Instron Corp) as previously described (23). Before testing, the left femur from 4-wk-old or 8-wk-old mice was hydrated in physiological saline (9 g NaCl/L) for 4 h at room temperature. During mechanical testing, femurs were fractured at the midshaft site. An identical protocol was used for offspring at 4 wk or at 8 wk of age. Internal controls were used during biomechanical testing to offset variations in each experiment through vehicle administration in the mother in the case of maternal supplementation studies (C57Bl/6J background) or littermate controls in the case of the *Gif*^−/−^_(F2)_ mice (C57Bl/6N background).

### Skeletal Muscle Analysis

Muscle samples were processed as described previously (24). Briefly, the tibialis anterior, extensor digitorum longs, gastrocnemius and soleus muscles were carefully dissected from mice hind limbs (8-wk-old B_12_-deficient *Gif*^−/−^_(F2:VEH)_, *Gif*^−/−^_(F2:B12)_, and WT littermates) and then immediately dipped into a freezing solution of isopentane suspended in liquid nitrogen for 10 s and stored at −80°C. Muscle samples were then cut in a cross-sectional orientation through the muscle midbelly in a −20°C cryostat-microtome (CM1850UV, Leica, UK) using a scalpel into two halves. One half was then embedded in the optimum cutting temperature compound (Qiagen) and mounted onto the microtome. Cross-sectional cryosections of 10-µm thickness were then taken, mounted onto glass slides (Thermo Fisher Scientific), and stored at −20°C for staining. Muscle samples were then subjected to ATPase staining (acid preincubation, pH 4.47) to distinguish between fiber types (25). Microscopic images of ATPase-stained sections were taken at ×5 and ×20 magnification. Muscle fiber traits were manually counted on ×5 images using ImageJ software (NIH–v. 1.43) and represented as soleus fiber numbers. Fiber cross-sectional areas (CSAs) were measured on ×20 images. For each fiber type, 25 measurements were taken to calculate the mean CSA of type I or type IIA fibers for that muscle.

### Measurement of Serum Osteocalcin and Deoxypyridinoline Levels

All mice were terminally bled through cardiac puncture under anesthesia at the time of euthanasia to withdraw blood in serum separating tubes (Microvette 500 Z-Gel). Blood was allowed to clot for 30 min and then centrifuged at 15,000 rpm for 10 min at 4°C to prepare serum. Serum was collected and stored at −80°C till analysis. Serum Osteocalcin and deoxypyridinoline levels were measured by ELISA assays as described previously (21).

### Molecular Assays

For RNA preparation, long bones were dissected free of surrounding tissues. Epiphyses were cut out and the bone marrow flushed. Three to four animals were analyzed independently. We used RT-PCR to analyze variations of gene expression between individual wild-type and mutant animals. RNA extraction, cDNA synthesis, and PCR amplification were performed using standard protocols as described previously (26). β-Actin amplification was used as an internal control. These genes were selected as markers of pre- or mature osteoblasts (*Runx2, Osx, Col1a1,* and *Ocn*) and differentiated osteoclasts (*Trap*). qPCR primer sequences used were as follows: *Ocn*F: TTTGTAGGCGGTCTTCAAGC; *Ocn*R: AAGCAGGAGGGCAATAAGGT; *Osx*F: ATGGCGTCCTCTCTGCTTG; *Osx*R: TGAAAGGTCAGCGTATGGCTT; *Runx2*F: TTCAACGATCTGAGATTTGTGGG; *Runx2*R: GGATGAGGAATGCGCCCTA; *Col1a1*F: GCTCCTCTTAGGGGCCACT; *Col1a1*R: CCACGTCTCACCATTGGGG; *Trap*F: CACTCCCACCCTGAGATTTGT; and *Trap*R: CATCGTCTGCACGGTTCTG.

### Statistical Analysis

Data are presented as the means ± SE, and statistical analyses were performed using the SAS System (v. 9.2, SAS Institute, Cary, NC). Statistical significance was declared at Body weight data were analyzed by PROC MIXED repeated-measures ANOVA (dam diet, pup diet, and time as main factors). All other measures were analyzed by general linear model two-way ANOVA (dam diet and pup diet as main factors), followed by Tukey’s pairwise multiple comparisons test to determine differences among groups. Outliers were identified by the use of Grubbs’ test (27).

## RESULTS

### Maternal B_12_ Supplementation during Gestation Increases Bone Mass Accrual of the Offspring

**Figure 1.**
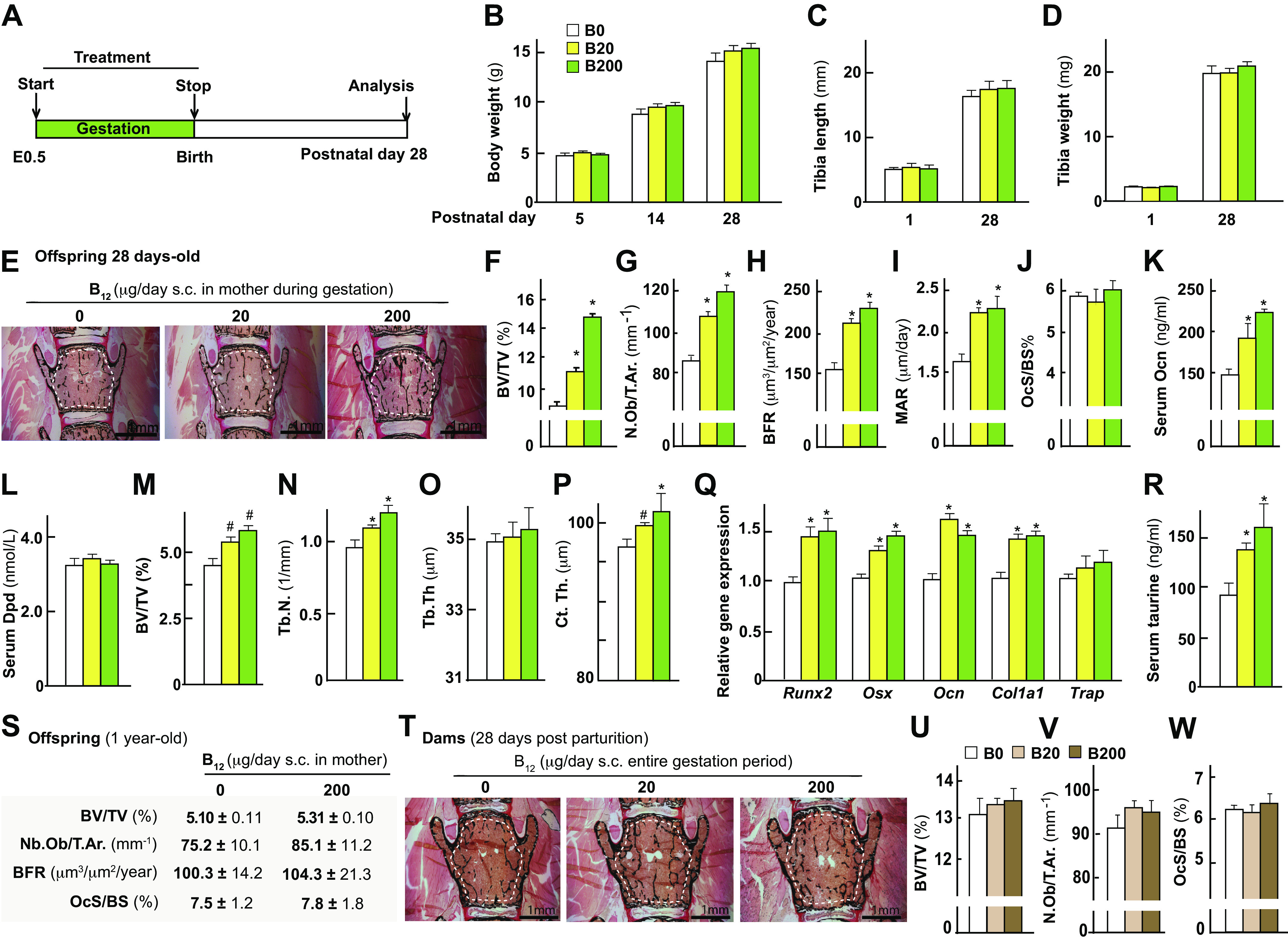
Maternal B_12_ supplementation in the wild-type mice during gestation increases bone mass accrual of the offspring during postnatal growth. *A*: schematic representation of the experimental protocol used. Body weight analysis (*B*), tibia length (*C*), tibia weight (*D*), representative images of Von Kossa/Van Gieson-stained vertebral sections (area used to measure BV/TV% is shown) (*E*), bone volume over total volume % (BV/TV%) in L4 vertebral sections of the offspring (*F*), osteoblast number per trabecular area (*G*), bone formation rate (*H*), and mineral apposition rate (*I*), osteoclast surface/bone surface % (*J*), serum osteocalcin levels (*K*), serum deoxypyridinoline (dpd; *L*) levels in offspring from mothers that received either vehicle (0) or B_12_ daily through subcutaneous injections at the dose of 20 or 200 μg/day during gestation from *embryonic day 0.5* to birth. Micro-CT analysis of tibia showing BV/TV% (*M*), trabecular numbers (*N*), trabecular thickness (*O*), and cortical thickness (*P*). Gene expression analysis of markers of osteoblast differentiation (*Q*) and serum taurine levels in offspring (*R*). *S*: histomorphometric analysis of L4 vertebra of offspring at 1 yr of age from mothers treated with either 0 or 200 μg/day dosage of B_12_ during gestation. Histological and histomorphometric analysis of L4 vertebra in mothers that received either vehicle (0) or B_12_ daily through subcutaneous injections at the dose of 20 or 200 μg/day during pregnancy: representative images of Von Kossa/Van Gieson-stained vertebral sections (area used to measure BV/TV% is shown) (*T*), BV/TV% (*U*), Osb.N./T.ar. (*V*), and Oc.S./BS% (*W*) is shown. Means ± SE is shown. *n* = 8–10 (*A*–*R*), *n* = 5 (*S*), *n* = 8 (*T*–*W*) female mice each group. *P < 0.05. #*P* < 0.01.

We first investigated the effect of B_12_ supplementation during gestation on early postnatal bone deposition in the offspring. For this purpose, we timed-mated 6-wk-old mothers (*n* = 5 each group) and treated them with either vehicle (B0), 20 (B20), or 200 (B200) μg of B_12_ daily through subcutaneous injections from *dpc 0.5* to *20.5*. Following delivery, mothers and offspring were left untreated after birth and euthanized after 4 wk (Fig. 1*A*). Body weight analysis from birth until euthanasia showed no significant change between offspring born from vehicle and B_12_-treated mothers (Fig. 1*B*). Likewise, there was no difference in the length or weight of the bones (tibia) in the offspring born from vehicle and B_12_-treated mothers at birth or at 4 wk of age (Fig. 1, *C* and *D*). Bone histological and histomorphometric analysis in the vertebra showed a dose-dependent increase in BV/TV% in offspring born from B_12_-treated mother [11.2 ± 0.4 (B20) and 14.3 ± 0.6 (B200)] compared with control offspring born from vehicle-treated mothers (8.1 ± 0.2) (Fig. 1, *E* and *F*). This increase in bone mass was due to an increase in osteoblast parameters (osteoblast numbers, bone formation rate, and mineral apposition rate) whereas osteoclast parameters [Osteoclast surface per bone surface (OcS/BS)%] were not affected in the offspring born from mothers that received B_12_ compared with offspring from vehicle-treated mothers (Fig. 1, *G*–*J*). The changes in bone formation and resorption were further confirmed through the measurement of serum Osteocalcin (Ocn) and deoxypyridinoline levels (Fig. 1, *K* and *L*). Micro-CT analysis of proximal tibia (long bone) showed a significant increase in BV/TV% in the B_12_-treated groups compared with the vehicle-treated offspring (Fig. 1*M*). This increase in BV/TV% was caused by an increase in trabecular numbers, whereas trabecular thickness was not affected (Fig. 1, *N* and *O*). Analysis of midshaft of the femur showed an increase in the cortical thickness in the B_12_-treated groups compared with vehicle-treated groups (Fig. 1*P*). Accordingly, and consistent with our earlier observations of a major effect of B_12_ on osteoblasts, but not osteoclasts markers of pre- and mature osteoblasts were increased in the bones of B_12_-treated groups compared with vehicle controls (Fig. 1*Q*). Because B_12_ is primarily stored in the liver and acts on osteoblasts through the regulation of liver taurine synthesis (17), we measured serum taurine levels as an indirect measure of B_12_ activity in the offspring. Serum taurine levels were significantly elevated in the offspring compared with the vehicle-treated controls (Fig. 1*R*). This latter result is consistent with our earlier observation that B_12_ indirectly acts on the osteoblasts through an increase in liver taurine synthesis (17). We next analyzed whether increase in bone mass that occurs in the offspring at 1 mo of age through maternal supplementation of B_12_ persists during aging in the offspring. Histological and histomorphometric analysis of vertebra of offspring at 1 yr of age from B_12_-supplemented mothers showed that their bone mass and cellular parameters were similar to the ones from vehicle-treated mothers (Fig. 1*S*). These results reveal that maternally derived B_12_ store is depleted during aging of the offspring and that maternally derived B_12_ does not affect age-induced changes in the bone in the offspring at 1 yr of age. The threshold of serum B_12_ required for long-term effect is perhaps larger and is currently not known. Given that a major change in bone mass occurs during the lactating period in the mothers, we next used the mothers postdelivery from the above experiment to analyze whether B_12_ administration during gestation had any effect on the lactation-induced changes in bone mass. Bone histological and histomorphometric analysis of the vehicle and B_12_-treated mothers showed no major change in the BV/TV%, osteoblast, or osteoclast parameters of B_12_-treated mothers [13.4 ± 0.3 (B20) and 13.5 ± 0.5 (B200)] compared with vehicle-treated control mothers (13.2 ± 0.8) (Fig. 1, *T–W*).

Together these results show that maternal supplementation of B_12_ impacts bone architecture in both the vertebra and long bone of the offspring at 4 wk of age, but not their mothers.

### B_12_ Deficiency Leads to Deterioration of Trabecular and Cortical Architecture of the Load-Bearing Long Bones

The maternal supplementation studies showed that an increase in maternal B_12_ during gestation is sufficient to increase bone mass in the offspring and raised the question of whether B_12_ deficiency in the mother leads to deterioration of bone quality. To test this contention, we next characterized structural changes in the bone and biomechanical properties of the long bone in the offspring following B_12_ deficiency using a mouse genetic model in which Gif protein that is essential for B_12_ transfer has been ablated. In this mouse model, we have earlier shown that B_12_ deficiency only precipitates in the *Gif*^−/−^_(F2)_ mice and not *Gif*^−/−^_(F1)_ mice due to a maternal transfer of B_12_ in the first generation (17). The *Gif*^−/−^_(F2)_ mice have normal skeletal mineralization at birth and have normal growth up until 21 days of age and the consequences of B_12_ deficiency on bone only precipitates in these mice postweaning (17). This B_12_ deficiency compromises the trabecular bone mass of the vertebra in *Gif*^−/−^_(F2)_ compared with littermate controls, and this deterioration in the vertebral bone mass can be reversed by a single injection of B_12_ during pregnancy in *Gif*^−/−^_(F1)_ females (17).

**Figure 2.**
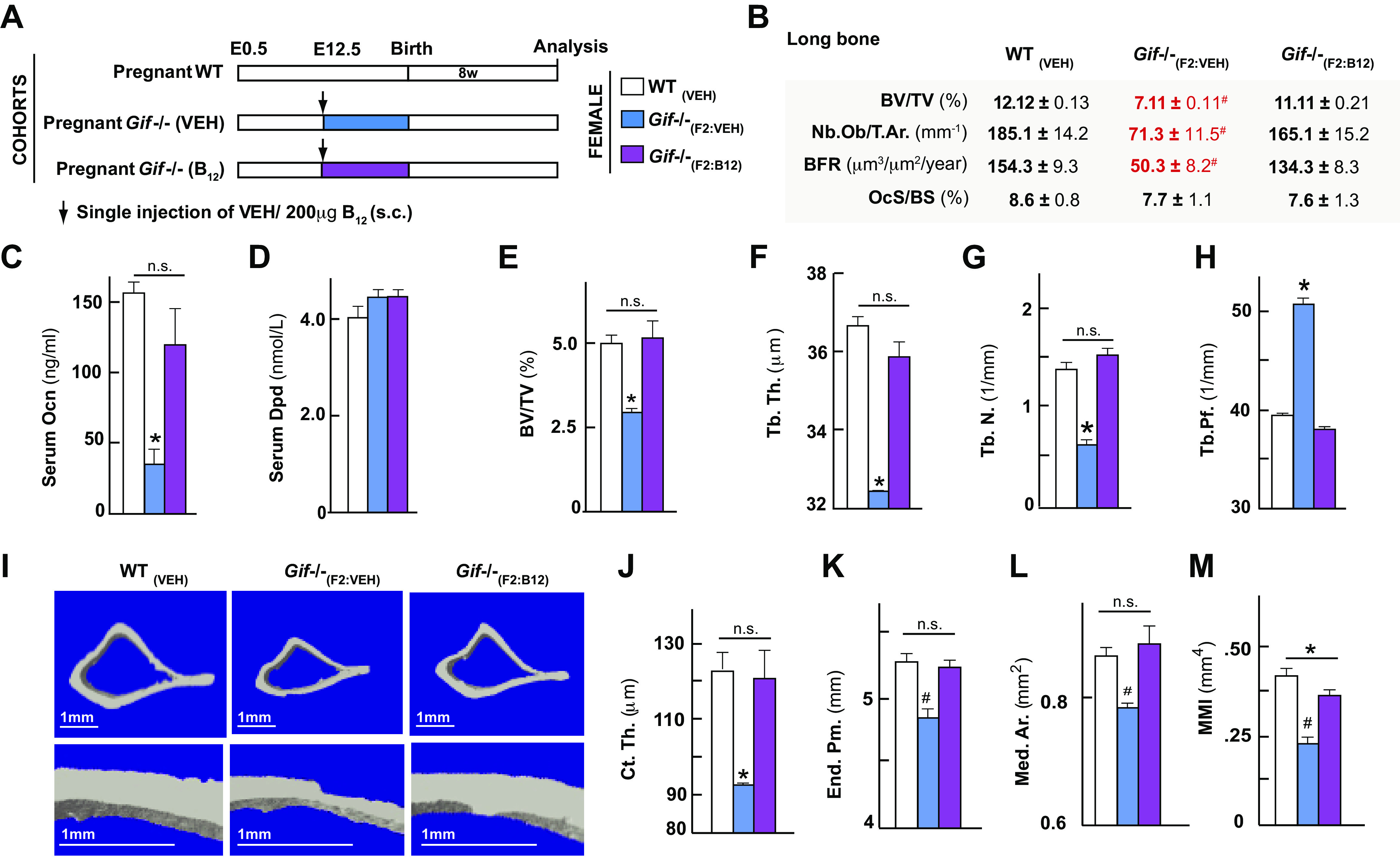
B_12_ deficiency compromises trabecular and cortical architecture in load bearing bones and a single injection of B_12_ in the mother rescues compromised bone architecture in the offspring. Schematic representation of the experimental protocol used (*A*), histological and histomorphometric analysis of the tibia (*B*), serum osteocalcin levels (*C*) and serum dpd levels (*D*) in 8-wk-old offspring born from mothers that received a single injection of vehicle or B_12_ at *embryonic day 12.5* are sown. Micro-CT analysis of the proximal tibia: bone volume/total volume (BV/TV)% (*E*), trabecular thickness (Tb.Th.) (*F*), trabecular number (Tb.N.) (*G*), trabecular patterning factor (Tb.Pf.) (*H*) is shown. *I*: representative micro-CT images of midshaft in tibia of 8-wk-old offspring born from mothers that received a single injection of vehicle or B_12_ at *embryonic day 12.5*. Cortical thickness (Ct.Th.) (*J*), endo-osteal perimeter (End. Pm.) (*K*), medullary area (Med. Ar.) (*L*), and moment of inertia (MMI; *M*) are shown. Means ± SE is shown. *n* = 8 (*B*–*D*), *n* = 10 (*E*–*M*) female mice each group. *P < 0.05. #*P* < 0.01.

Using WT and *Gif*^−/−^_(F2)_ mice, we next characterized B_12_ deficiency-induced cortical and trabecular changes in the long bones. To this end, timed-mated pregnant WT or *Gif*^−/−^_(F1)_ females were given either vehicle or a single injection of 200 µg of CN-B_12_ (sc) on 12.5 days postcoitum (dpc), and their offspring are labeled as *Gif*^−/−^_(F2:VEH)_ or *Gif*^−/−^_(F2:B12)_ mice (Fig. 2*A*). Analysis of serum levels of B_12_ in these mice revealed that although B_12_ levels remained below the detection limit of the assay (<45 ng/mL) in *Gif*^−/−^_(F2:VEH)_ mice, the levels in *Gif*^−/−^_(F2:B12)_ mice were 150.1 ± 30.1 ng/mL compared with WT controls. Bone histological and histomorphometric analysis of the proximal femur showed that BV/TV% was significantly reduced in the *Gif*^−/−^_(F2:VEH)_ mice compared with WT control mice, but the levels were similar between *Gif*^−/−^_(F2:B12)_ and WT mice (Fig. 2*B*). This increase in bone mass in *Gif*^−/−^_(F2:B12)_ was caused by a major increase in osteoblast numbers and bone formation rate while bone resorption remained relatively unaffected compared with WT mice (Fig. 2*B*). The changes in bone formation and resorption were further confirmed through the measurement of serum Osteocalcin (Ocn) levels and serum deoxypyridinoline levels (Fig. 2, *C* and *D*). Micro-CT analysis of trabecular parameters in the proximal tibia from WT and *Gif*^−/−^_(F2:VEH)_ and *Gif*^−/−^_(F2:B12)_ mice revealed a significant decrease in bone volume/total volume (BV/TV) %, trabecular number (Tb.N.), trabecular thickness (Tb.Th.), and high trabecular patterning factor (Tb.Pf.) (Fig. 2, *E*–*H*) in the *Gif*^−/−^_(F2:VEH)_ mice compared with WT mice. These changes in trabecular parameters contributed toward an increase in bone surface per bone volume in *Gif*^−/−^_(F2:VEH)_ bones (data not shown). We next determined cortical bone parameters in the midshaft of the tibia. The *Gif*^−/−^_(F2:VEH)_ mice had a severe decrease in cortical thickness (Ct.Th.) confirming our earlier observations (Fig. 2, *I* and *J*) (17). Long bones of offspring from vehicle-treated B_12_-deficient mothers also displayed a decrease in endosteal perimeter (End.Pm.) and medullary area (Med.Ar.) compared with the controls (Fig. 2, *K* and *L*). Consequently, *Gif*^−/−^_(F2:VEH)_ mice displayed a reduction in cortical cross-sectional moment of inertia reflecting toward a reduced bone strength (Fig. 2*M*). Analysis of *Gif*^−/−^_(F2:B12)_ mice showed no significant change in the trabecular and cortical parameters tested compared with the WT mice demonstrating that a single injection of B_12_ in the mother is sufficient to revert long bone architectural deterioration in the offspring (Fig. 2, *A–M*). Together these results show that B_12_ deficiency in the mother leads to changes in both the trabecular and cortical compartments in long bones of the B_12_-deficient *Gif*^−/−^_(F2:VEH)_ offspring, which can be completely reversed by a single injection of B_12_ to the mother.

### Effect of Changes in B_12_ Levels on Bone Biomechanical Strength

**Figure 3.**
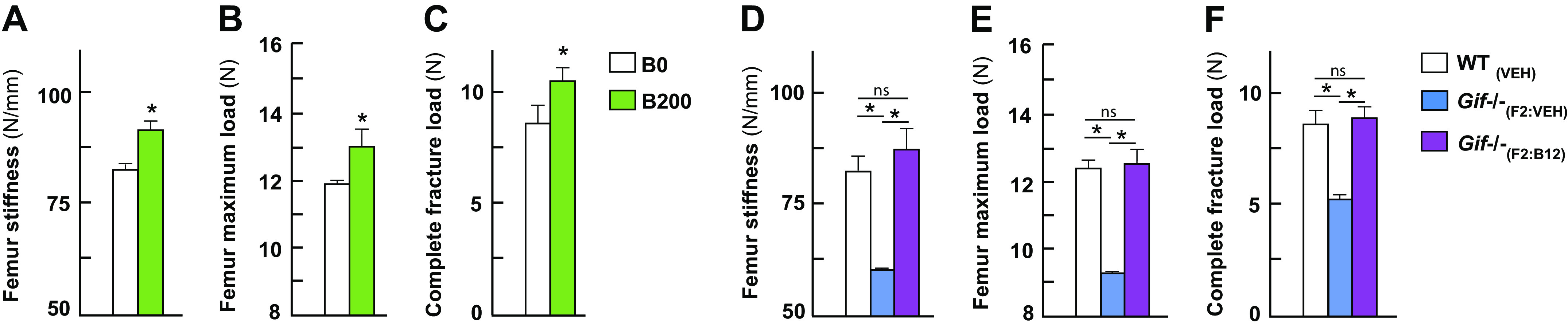
B_12_ supplementation and B_12_ deficiency during gestation affects biomechanical strength of the load-bearing bones in the offspring. Analysis of biomechanical properties in the femur of 4-wk-old offspring from mothers (C57BL/6J genetic background) that received either vehicle (0) or B_12_ daily through subcutaneous injections at the dose of 200 μg/day during gestation from *embryonic day 0.5* to birth. Three-point bend test femur stiffness (*A*), femur maximum load (*B*), and complete fracture load of the long bone (femur) (*C*) are shown. Biomechanical properties of the long bone of 8-wk-old offspring born from *Gif*^+/+^ or *Gif*^−/−^ mothers (C57BL/6N genetic background) that received a single injection of vehicle or B_12_ at *embryonic day 12.5*. Three-point bend test femur stiffness (*D*), femur maximum load (*E*), and complete fracture load of the long bone (femur) (*F*) are shown. Means ± SE is shown. *n* = 8 (*A*–*C*), *n* = 9 (*D*–*F*) female mice each group. *P < 0.05.

Bone biomechanical property depends on the amount of bone present, the spatial distribution of the bone, and the cortical and trabecular microarchitecture of different bone compartments. The observed increase in bone architecture especially in the cortical bone in the offspring born from the mothers that received B_12_ compared with the ones that received vehicle indicated toward an improved bone quality. To determine whether bone biomechanical properties were indeed improved, we next measured biomechanical properties of femur samples of offspring from mothers treated with vehicle (B0) and 200 (B200) μg of B_12_ through a three-point bending test. The three-point bending test is the most common method performed to determine maximal load and stiffness, two surrogates of bone quality, of the femur when subjected to an external load applied perpendicularly to the longitudinal axis. Both femur stiffness and maximal load were significantly increased in the B200 group compared with the B0 group (Fig. 3, *A*–*C*). Thus, B_12_ supplementation during gestation leads to increased bone quality in the offspring.

We next investigated the effect of maternal B_12_ deficiency on the biomechanical properties of bones in the offspring. Both femur stiffness and maximal load were significantly decreased in *Gif*^−/−^_(VEH)_ mice and restored to values seen in WT mice by a single B_12_ injection to the mother on an embryonic *day 12.5* during gestation (200 µg sc) (Fig. 3, *D*–*F*). Thus, B_12_ deficiency in *Gif*^−/−^_(VEH)_ mice leads to a decrease in bone quality and this deterioration can be completely rescued in the *Gif*^−/−^_(B12)_ mice to the levels seen in the WT mice.

### B_12_ Deficiency Does Not Affect Overtly Skeletal Muscle

**Figure 4.**
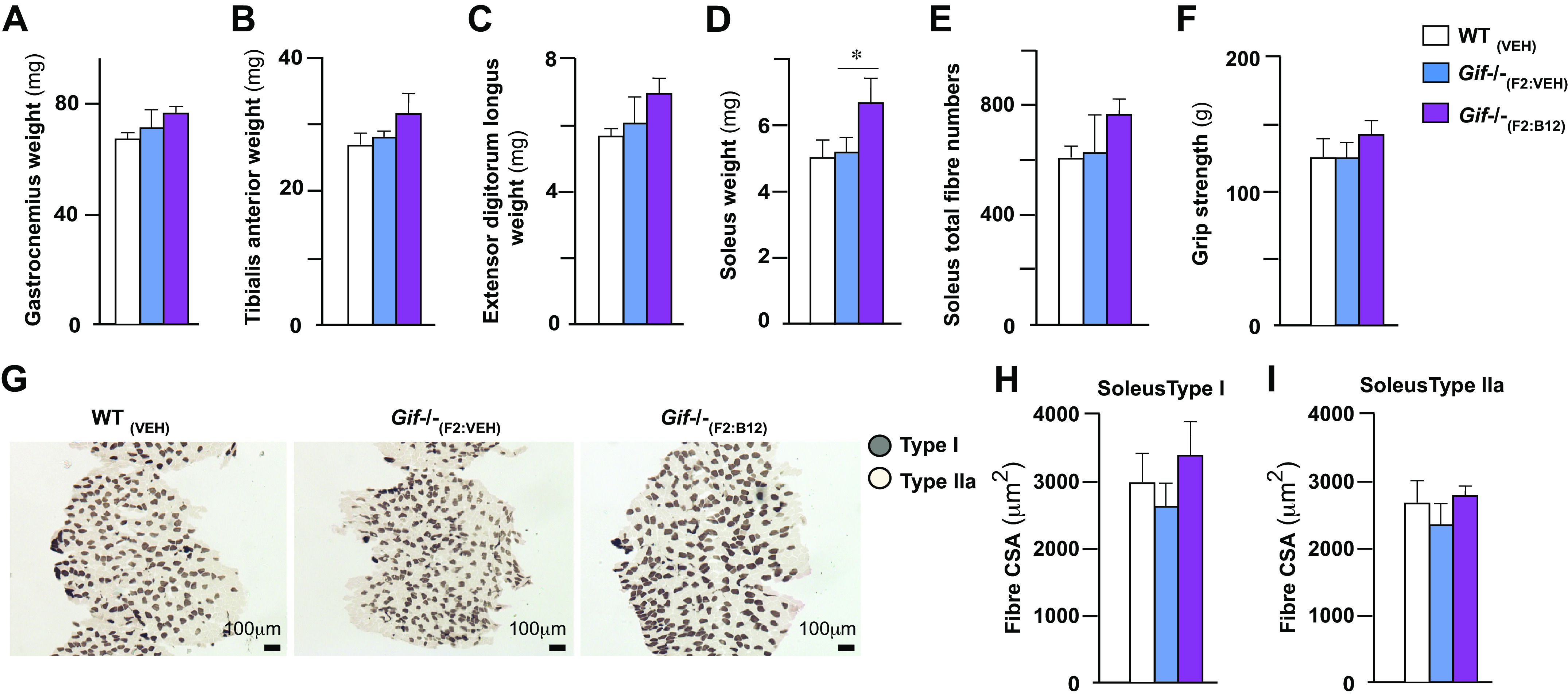
Effect of B_12_ deficiency and supplementation on the skeletal muscle. Gastrocnemius weight (*A*), tibialis anterior weight (*B*), extensor digitorum longus weight (*C*), soleus muscle weight (*D*), soleus total fiber numbers (*E*), grip strength (*F*), representative image of soleus muscle fiber histology with ATPase staining showing distribution of soleus muscle fibers (*G*), soleus Type I (*H*), and soleus Type IIa fibers cross-sectional area (CSA; *I*) in 8-wk-old offspring born from mothers that received a single injection of vehicle or B_12_ at *embryonic day 12.5* are shown. Means ± SE is shown. *n* = 3–10 mice each group. *P < 0.05.

We next tested whether B_12_ deficiency in 8-wk-old mice affects any aspect of skeletal muscle parameters. To test whether B_12_ is a regulator of muscle mass in growing mice, we analyzed the weight of hind limb muscles (tibialis anterior, gastrocnemius, soleus, extensor digitorum longus, and soleus) in 8-wk-old B_12_-deficient *Gif*^−/−^_(F2:VEH)_, *Gif*^−/−^_(F2:B12)_, and WT_(VEH)_ littermates. Given lower body weights in the *Gif*^−/−^_(F2:VEH)_ mice compared with the other two groups, it was considered as a variable when interpreting the muscle data. This analysis revealed neither a difference in any of the hind limb muscle weights or soleus total fiber numbers between wild-type and the *Gif*^−/−^ genotypes (Fig. 4, *A*–*E*). Consistent with no change in muscle weights of different muscle types analyzed in the hind limb grip strength was similar between wild-type and the *Gif*^−/−^ genotypes (Fig. 4*F*). We next performed ATPase staining on the soleus muscle sections to determine whether changes in B_12_ levels in the offspring had any effect on the fiber-type distribution of the soleus muscle (Fig. 4*G*). This analysis revealed no difference in either the Type I or Type IIa muscle fibers (Fig. 4, *H* and *I*). Together these analyses showed that B_12_ deficiency in the mother does not affect the muscle weight or the fiber type distribution in the offspring at 8 wk of age.

## DISCUSSION

Deterioration of bone quality due to aging, genetic, or environmental factors increases the risk of fractures in humans (28, 29), yet the molecules underlying these processes remain unclear. Our findings illustrate that maternally derived B_12_ is a critical determinant of bone architecture and biomechanical properties and identify a new molecule that regulates bone quality. Although doing so we generated a mouse model of B_12_ deficiency that can now be used to better define the molecular and cellular pathophysiology in different bone compartments observed in humans with B_12_ deficiency.

This study is the first to demonstrate changes in bone architecture and quality following B_12_ deficiency as a consequence of loss of gastric intrinsic factor expression in the stomach. In our earlier studies, we had shown that at the level of histology there is a change in the bone mass in the vertebra. We now extend these findings to the three-dimensional (3-D) level architecture and cellular parameters of the long bone. B_12_ regulates bone quality by affecting positively the cortical and trabecular bone compartments, the two major types of bones that regulate the risk of fractures. In the trabecular compartment, B_12_ deficiency decreased the thickness, numbers, and connectivity of trabeculae, whereas in the cortical compartment, it had a profound influence on the cortical thickness and porosity. The architectural deterioration in the cortical and trabecular compartments led to a major decrease in the whole body bone mineral density and content and consequently to a decrease in biomechanical properties of the long bones. Our studies also show that B_12_ supplementation of the mother during gestation affects the bone accrual of the offspring postnatally during peripubertal growth. However, this maternally derived B_12_ which is stored in the liver and is recycled gets depleted over time and by 1 yr of age offspring from vehicle- or B_12_-treated mothers have similar bone mass and cellular parameters. This latter result is consistent with our earlier observation wherein we showed that offspring that are unable to absorb B_12_ on their own but received B_12_ during gestation from their mother are protected from deleterious consequences of B_12_ deficiency during early postnatal life but develop low bone mass at 1 yr of age (17).

Our studies showing that only the second-generation *Gif*^−/−^ mice and not the first-generation *Gif* animals show a decrease in long bone density and quality provide a plausible explanation for earlier studies that have shown either no change or a poor effects of acute dietary deficiency of B_12_ on bone mass (30) or studied the effect of feeding a B_12_-free diet (BFD) for 12 wk on bone mineral density and bone architecture parameters in rats. These studies did not observe any significant change in B.Ar./T.Ar., OC, and CTX did not differ between BFD and control animals despite the fact that homocysteine was elevated in the BFD-fed animals. Thus, our study where we observed a decrease in bone mass only after the F1*Gif* mice were aged for 1 yr and that of Hermann et al. (30), suggest that only long-term and not a short-term deficiency of B_12_ leads to deterioration in the bone architecture and quality at least in rodents. In our studies, the effect of in vivo maternal supplementation on the bone was associated with an increase in osteoblast parameters, whereas the osteoclast compartment was overall unchanged. Given that osteoblasts and osteoclasts work in coordination, this is a surprising observation, but it is consistent with our earlier studies looking at the effect of B_12_ deficiency on bone formation (17). There are however many physiological and pathological situations in which osteoblast and osteoclast functions are uncoupled, and one such example is neural regulation of bone mass (31–33).

In contrast to the bone, the B_12_ deficiency in *Gif*^−/−^_(F2)_ had little effect on muscle size or functional properties. In the *Gif*^−/−^_(F2:B12)_ offspring born from B_12_-supplemented *Gif*^−/−^_(F1)_ mothers, there is an increase in Sol weight significantly and there is a trend toward an increase in grip strength and soleus fiber numbers compared with vehicle-treated *Gif*^−/−^_(F2:VEH)_ offspring. However, this might be explained by an overall body weight difference in the two cohorts so generally changes in B_12_ metabolism have at best small effect on skeletal muscle mass and function. It has been shown earlier that patients with sarcopenic have lower levels of B_12_, but the causal relationship was not clear (34). Our studies highlight that in severe B_12_-deficient states, at least in the mouse model, it does not affect muscle architecture or function. However, the mechanisms in humans and mouse for muscle regulation through B_12_ could be different. Also, it is possible that severe B_12_ deficiency, as present in our mouse genetic model, could lead to activation of compensatory mechanisms accounting for no major changes in its structure or function. Further studies are needed to explore these avenues.

## PERSPECTIVES AND SIGNIFICANCE

Maternally derived factors regulate a variety of physiological processes in the offspring yet they remain poorly understood. In the present study, we report the effects of changes in levels of B_12_ in the mother on the bone mass and mechanical strength and muscle mass and strength. We show that the B_12_ supplementation in the mother increases bone mass and mechanical strength. Conversely, B_12_ deficiency negatively affects bone mass and compromises bone quality. However, maternally derived B_12_ does not affect muscle fiber mass and numbers and grip strength, thus emphasizing the fact that B_12_ of maternal origin affects different musculoskeletal compartments differently. Our demonstration that B_12_ levels in the mother are determinant of bone mass and mechanical strength in the offspring provides evidence that mothers equip offspring by providing nutrient depots for challenges that they may face postnatally. An important role of B_12_ in building and maintaining bone mass and strength during a peripubertal period in the offspring suggests that modulation of B_12_ levels or activity in the mother has the potential to enhance bone mass during early growth years in the offspring.

## GRANTS

This work was supported by Wellcome Trust Grant 098051 and the National Institute of Immunology core Grant (to V. K. Y.).

## DISCLOSURES

No conflicts of interest, financial or otherwise, are declared by the authors.

## AUTHOR CONTRIBUTIONS

V.K.Y. conceived and designed research; P.S., S.T., B.Z., A.D.M., V.D.M., H.W., and V.K.Y. performed experiments; P.S., B.Z., A.D.M., V.D.M, H.W., and V.K.Y. analyzed data; P.S., A.D.M., V.D.M., H.W., X.E.G., and V.K.Y. interpreted results of experiments; P.S., A.D.M., V.D.M., H.W., X.E.G., and V.K.Y. prepared figures; P.S., H.W., X.E.G., A.K.P., and V.K.Y. drafted manuscript; P.S., H.W., and X.E.G. edited and revised manuscript; P.S., H.W., X.E.G., and V.K.Y. approved final version of manuscript.
